# Considering behaviour to ensure the success of a disease control strategy

**DOI:** 10.1098/rsos.170721

**Published:** 2017-12-06

**Authors:** Christopher Finn McQuaid, Christopher Aidan Gilligan, Frank van den Bosch

**Affiliations:** 1 Computational and Systems Biology, Rothamsted Research, West Common, Harpenden AL5 2JQ, UK; 2 Department of Plant Sciences, University of Cambridge, Downing Street, Cambridge CB2 3EA, UK

**Keywords:** behavioural model, clean seed system, cassava brown streak

## Abstract

The success or failure of a disease control strategy can be significantly affected by the behaviour of individual agents involved, influencing the effectiveness of disease control, its cost and sustainability. This behaviour has rarely been considered in agricultural systems, where there is significant opportunity for impact. Efforts to increase the adoption of control while decreasing oscillations in adoption and yield, particularly through the administration of subsidies, could increase the effectiveness of interventions. We study individual behaviour for the deployment of clean seed systems to control cassava brown streak disease in East Africa, noting that high disease pressure is important to stimulate grower demand of the control strategy. We show that it is not necessary to invest heavily in formal promotional or educational campaigns, as word-of-mouth is often sufficient to endorse the system. At the same time, for improved planting material to have an impact on increasing yields, it needs to be of a sufficient standard to restrict epidemic spread significantly. Finally, even a simple subsidy of clean planting material may be effective in disease control, as well as reducing oscillations in adoption, as long as it reaches a range of different users every season.

## 1. Background

The adoption of a costly control strategy for disease can be viewed as a public goods problem, and has been studied in many systems, particularly for vaccination (e.g. [1]; for a review, see [2]). Oscillations in adoption of the control strategy are often observed; waves of infection and subsequent adoption are followed by a decrease in adoption as infection declines and control is deemed too costly in the absence, followed by a resurgence, of infection (e.g. [3,4]). Cost thresholds, above which adoption decreases dramatically as very few individuals apply the control, may also reduce the success of a disease control strategy for structured populations [1]. The economic and social consequences of this instability may make control strategies unsuitable or unsustainable, as practitioners and businesses fail to cope with constant changes in demand. It is therefore important to integrate considerations of grower behaviour with those of disease to determine policy (e.g. [5,6]). Such integration has seldom been done for agriculture (although see [4,7]), but which could benefit greatly from the increased stability and savings incurred.

A seed system distributes fresh (disease-free and high yielding), improved (resistant or tolerant of disease) planting material (‘clean seed’) to growers to reduce crop losses. These systems are frequently used as a control measure for disease. Use of clean planting material reduces disease within a field, but to reduce reinfection from neighbouring fields several users in an area are required to act collectively. However, non-participating growers may also obtain herd immunity (whereby high levels of control in the population decrease disease to such an extent that even growers failing to control for the disease are protected by a general reduction in inoculum pressure from neighbours' fields) (e.g. [4]). The increased costs of clean seed inhibit adoption. Growers are then likely to choose what they perceive to be the most economical approach [4]. Additionally, lack of knowledge about the disease (its presence and dispersal mechanisms), a preference for local landraces (due to issues such as trust in their quality and taste), or a lack of access (reduced availability and quality of planting material, or the financial means to purchase clean seed) may reduce adoption (e.g. [8–10]). Accordingly, we want to know how to increase the adoption of planting material from these seed systems (Q1), how to avoid potential pitfalls such as oscillations in adoption (Q2) and how best to implement active interventions through subsidies (Q3).

Cassava, a key tuberous root crop for subsistence growers in sub-Saharan Africa, is an ideal system for which to study these questions. Cassava brown streak disease is a viral disease found in East Africa that reduces the yield of cassava through reduction in plant growth and necrosis of the tubers [11] and it is increasingly becoming a problem across the region [12]. The viruses that cause the disease are transmitted by a whitefly vector and may also be propagated through infected planting material [13–15], suggesting that clean planting material may be beneficial for disease control. Indeed, a previous model has examined the spread of the pathogen across a landscape and the effect of a seed system in reducing spread [16]. However, that model [16] did not account for the effects of grower behaviour. Currently only a few cassava growers use certified clean planting material due to low availability, in itself due to low multiplication rates, bulkiness and perishability of cassava planting material ([8], although see [17]). Growers are also unwilling or ignorant of the need to remove infected plants, and are constrained by the costs such removal incurs due to decreased yields [14,18–21].

A previous project, the Great Lakes Cassava Initiative, piloted distribution of small quantities of clean planting material. A current Bill and Melinda Gates Foundation-funded project and a separate organization, the Mennonite Economic Development Associates, are aiming to establish large-scale seed systems in Uganda and Tanzania. Here we investigate the effect of grower behaviour on such systems, demonstrating how to increase their success and ensure their sustainability in terms of disease control.

## 2. Methods

The dynamics for plant and vector populations are taken from McQuaid *et al.* [16], and are outlined below. These dynamics describe the dispersal of the pathogen across the district, through both trade and the dispersal of viruliferous whitefly, as well as within individual fields through a mean-field model. The use of a clean seed system affects infection in the planting material that a grower chooses to replant.

The grower behaviour model is layered on top of this model, where each grower makes a seasonal decision on whether to adopt the control strategy. The effect of this decision then feeds back into the dynamics of the disease itself. The decision is based on the relative ‘reward’ that the grower has observed for each strategy (adoption or non-adoption of the control) in the previous season among either her neighbours, trade partners or across the district as a whole, compared with the reward that she obtained in her field. This reward is a combination of the total yield obtained and the costs incurred in purchasing planting material. We outline the model below, which is simulated in Matlab, where further details of the disease dynamics model can be found in McQuaid *et al.* [16]. We investigate the sensitivity of the model to the cultural setting (the nature of farming and trading) and grower behaviour, with a discussion of the disease model to be found in McQuaid *et al.* [16], noting that in the absence of a grower behaviour model the system tends towards a steady state containing both infected and susceptible plants, while in the absence of control the disease becomes ubiquitous. We vary parameters that describe the three different components of the system: the farming system and disease presence in the area, the clean seed material and how it is introduced, and the grower behaviour in response to this introduction. In this way we can compare the outcomes we might expect in different areas with different seed systems, evaluating the influence that grower behaviour has on our results

### 2.1. Population dynamics

#### 2.1.1. Within season

For each field *i* we consider uninfected (*S_i_*), latently infected (*L_i_*) and infectious and symptomatic (*I_i_*) plants and infectious vectors (*V_i_*, with a total whitefly vector population given by *W*). For fields *i* = 1 … *N*, we consider

2.1 $$\begin{array}{rl} \frac{\text{d}S_{i}}{\text{d}t} & =\mu \left(1-q_{i}\overset{N}{\underset{j=1}{\sum }}\tau_{ij}\frac{C_{j}}{P_{j}}\right)-hS_{i}-\beta_{p}S_{i}V_{i},\\ \frac{\text{d}L_{i}}{\text{d}t} & =\mu q_{i}\overset{N}{\underset{j=1}{\sum }}\tau_{ij}\frac{C_{j}}{P_{j}}+\beta_{p}S_{i}V_{i}-(h+\gamma_{p})L_{i},\\ \frac{\text{d}I_{i}}{\text{d}t} & =\gamma_{p}L_{i}-(h+g)I_{i}\\ \text{and}\frac{\text{d}V_{i}}{\text{d}t} & =\beta_{v}(I_{i}+R)\cdot (W-V_{i})-(\lambda +\omega )V_{i}+m\left(\overset{N}{\underset{j=1,j\neq i}{\sum }}\delta_{ij}V_{j}-V_{i}\right). \end{array}\}$$

Plants are harvested at rate *h* and additional plants may be replanted at rate *µ*. Replanting occurs with planting material obtained at the beginning of a season, where we consider commercial (seasonal harvesting and replanting events), subsistence (continuous harvesting throughout the season with annual replanting events) and casual (continuous harvesting and replanting with original material throughout the season) growers.

Plants may additionally be infected through contact with viruliferous vectors, which infect uninfected plants at density-dependent rate *β*_p_. Resistant plants are infected at a lower infection rate *β*_p_. Once infected, latently infected plants progress to a fully infectious state at rate *γ*_p_, when they may be rogued at rate *g*.

In terms of the vector, uninfected vectors (*W* − *V_i_*) become infected at density-dependent rate *β*_v_ through contact with infectious plants in the same field or a reservoir host (at density *R*). The vector loses this infectivity at rate *λ*, and dies at rate *ω*. Vector between-field dispersal, where we assume emigration and immigration rates are the same, occurs at rate *m*. We assume this dispersal is constant for emigration, while for immigration into field *i* we sum dispersal of infectious vectors from each field *j* to field *i*. This is given for Euclidean distance *d_ij_* between the centres of the pair of fields by the probability density dispersal function $\delta_{ij}=(A\alpha^{2}\text{/}2\pi ){\text{e}}^{-\alpha d_{ij}}$, for attractive area *A* (where the dispersal probability at every point in the field is equal) and mean distance of dispersal 1/*α*.

#### 2.1.2. Between seasons

Replanting occurs with planting material obtained either from the grower's own field (*q_i_* = 1, with probability *b*_c_ if not using a clean seed system) or through trade with the owner of another field (*q_i_* = 1, with probability 1 − *b*_c_ if not using a clean seed system), or from a clean seed system (*q_i_* = 0). If the former, the proportion of cuttings in field *i* obtained from every field *j* is given by *τ_ij_*, so that $\sum_{j=1}^{N}\tau_{ij}=1$. For trade with neighbouring fields, a grower at field *i* trades with a number of neighbours up to a given maximum *b*_s_, where grower *j* is chosen with probability $\rho_{ij}=r{\text{e}}^{-d_{ij}/d_{\max}}$ for *d*_max_ the maximum distance between any two fields and random, uniformly selected variable r∈[0,1]. This latter variable *r* adds stochasticity to the trade process, ensuring that growers do not simply trade with their nearest neighbours. Growers may remain loyal to the same suppliers each season, or may alter suppliers, where a given number of growers *b*_l_ across the system are loyal. We note that for each pair of fields *i* and *j*, the probability of trade *ρ_ij_* is unrelated to the quantity of trade *τ_ij_*, although *τ_ij_* > 0 if and only if *ρ_ij_* > 0. The level of infection in the planting material obtained from field *j* is determined by $P_{j}=\int_{0}^{300}h(S_{j}+\eta L_{j})\;\text{d}t+(S_{j}+\eta L_{j}){|}_{300}$ and $C_{j}=\int_{0}^{300}h((1-\eta )L_{j}+I_{j})\;\text{d}t+((1-\eta )L_{j}+I_{j}){|}_{300}$, the total number of uninfected and infected plants respectively harvested from field *j* in the previous (300 day) season, either during the season at rate *h* or at the end of the season where all remaining plants are harvested. These are constant within a season, based on the dynamics of the previous season, but vary between seasons. Latently infected harvested plants may also have sufficiently low viral load as to undergo reversion at rate *η*, while infectious plants may be successfully identified as such and discarded with likelihood given by cutting selection parameter *ξ*. In this way, the proportion of infected cuttings replanted depends on whether or not the grower is using planting material directly obtained through a clean seed system, and the incidence of infection in planting material that the grower has collected both from her own and her neighbour's fields.

### 2.2. Grower behaviour

#### 2.2.1. Between seasons

The epidemiological model is continuous within a fixed duration season, with an annual discrete complete harvesting and replanting event. The behavioural model and the epidemiological model are then linked through an annual decision by the growers on whether or not to alter their choice of replanting strategy (affecting *q_i_* above), based on the reward that each strategy is perceived to produce. This follows the methods of Milne *et al.* [4]. A detailed analysis of the effect of parameter *q_i_* is included in McQuaid *et al.* [16]; in essence, as would be expected when clean seed is used there is less disease in a field. The incidence across the region then quickly equilibrates, where the choice of growers that use clean seed (i.e. for whom *q_i_* = 1) affects the equilibrium disease level. In particular, the spatial clustering of users affects results, as does the consistency of those users between seasons.

Decision-making for a grower takes place at the beginning of every season, and is therefore not concurrent to the disease spread model. A grower changes strategy with probability

2.2 $$\begin{array}{l} \sigma =1-{\text{e}}^{-\theta (\phi_{A}-\phi_{F}+\kappa )}\;\;\text{when}\;\;\phi_{A}-\phi_{F}+\kappa >0\\ \text{and}\;\;\psi \;\text{otherwise}. \end{array}$$

Here *θ* measures the responsiveness of the grower to loss, while *ϕ*_F_ is the reward that the grower obtained in the previous season and *ϕ*_A_ is the reward that the grower perceived among those of her farmer's group, trade partners or the general population who used the alternative strategy. Parameter *κ* measures the likelihood of a grower to conform to the strategy of their neighbours (*κ* > 0) or to stubbornly persist with their own strategy (*κ* < 0), in either case regardless of the actual reward both strategies offer (see (2.1)). We also note that there is a probability of switching strategy even when the alternative results in lower levels of reward, given by contrariness *ψ*, simulating irrational choices by growers as well as accounting for ignorance of the cause of the disease.

Reward is measured, for strategy *s* (either using a clean seed system or not), as

2.3 $$\phi_{s}=Y_{s}p-c_{s},$$

where *Y*_s_ is the proportion of yield that a grower using that strategy obtained in the previous season, *p* is the selling price of the crop that could have been obtained from an uninfected field, and *c*_s_ is the cost that using the strategy incurs. Yield, given by the harvest taken over the season including the final harvest, is $Y=\int_{0}^{300}h(S_{j}+L_{j}+\iota I_{j})\;\text{d}t+(S_{j}+L_{j}+\iota I_{j}){|}_{300}/\int_{0}^{300}h(S_{j}+L_{j}+I_{j})\;\text{d}t+(S_{j}+L_{j}+I_{j}){|}_{300}$, where the yield of latently infected plants is unaffected by disease, while only a proportion (*ι*) of infectious plants are usable. Tolerant plants have a higher useable portion *ι*. All fields are assumed to be identical in size and are therefore comparable, where growers receive information on relative yields from their farmers group of neighbouring growers with probability *r*_l_, their trade suppliers with probability *r*_t_ or across the district as a whole through extension workers with probability *r*_d_.

We initially presume that 70% of fields are infected with approximately 100% incidence (T. Alicai, 11 May 2015, personal communication) and 10% of growers use the clean seed system. See table 1 for default parameter values and box 1 for relevant terminology, where more details on parameter estimation may be found in McQuaid *et al.* [16].

Box 1. **Grower behaviour terminology.**

1. *Conformism*—the tendency to adopt the strategy of one's neighbours irrespective of the rewards different strategies display.
2. *Stubbornness*—opposite of conformism, the tendency to stick with one's current strategy irrespective of the reward displayed.
3. *Responsiveness*—the tendency to change strategies when there is only a small change in rewards.
4. *Contrariness*—the tendency to adopt a strategy that displays less reward than the alternative.

**Table 1.** Model parameters and default values. For simplicity, we assume that there is one growing season of 300 days per year, ignoring the initial two months of the season when there is no foliage and whitefly cannot transmit the pathogen, although we note that in reality there may be some transmission between 1 and 2 months. We assume that the majority of growers are subsistence farmers, as is often the case. This implies that fields are harvested continuously at a constant rate over a period of months during the season, commencing after the first two months, with one replanting event at the start of each season (*μ* = 0, *h* > 0). The size of a cassava field is taken to be 1.5 hectares (e.g. [22]), and we presume an increase in affinity for whitefly of cassava fields over other areas. We presume that whitefly are a third again as likely to land on a cassava field as on a bare patch of land. We base the cassava field density of our model on the area of cassava harvested in Nakasongola district, Uganda (10 000 hectares, http://kids.fao.org/agromaps/), and hence consider 6000 cassava fields. Whitefly migration is calculated from Riis & Nachman [23], using the total population to find the immigration rate at equilibrium, which we presume to be identical to emigration. To simulate the dispersal of the vector we use data from Isaacs & Byrne [24] and Byrne *et al.* [25] for the dispersal of the sweet potato whitefly (on average, 50–700 m). We do not include the long-distance dispersal of whitefly that Byrne *et al.* [25] observe in a second peak of migration, as this is not consistent with the exponential dispersal kernel that we have assumed. More importantly, however, whitefly may not remain infectious, or even survive, for the duration of these journeys [15,26,27]. See McQuaid *et al.* [16] for further details. For harvesting and replanting, one example of the maximum potential yield in Uganda is UGX 1 200 000, while the cost to growers using certified clean planting material is UGX 300 000 compared to UGX 0 for those that obtain planting material through the recycling of cuttings. It is the ratio of the maximum potential yield to the cost of technology that is important, which from the above is taken to be 1 : 0.25 : 0, although we vary this to consider other systems. We estimate the responsiveness of growers to loss from the known adoption of certified seed and seed systems for other crops by growers in East Africa (roughly 5–15%, see [28–31]) and the maximum potential benefit of using certified clean planting material (0.01–0.28). The latter is calculated from equation (2.3) and the equilibrium yield (approx. 100% for users of the clean seed system, 47–74% for nonusers) from equation (2.1) when 5–15% of growers dispersed across the landscape use the clean seed system, where the users vary each season. Solving equation (2.2) using these values gives the range 0.18 ≤ *θ* ≤ 16.25, where we pick *θ* from this range. We choose stubbornness *κ* to be of a similar order of magnitude, with a default value of zero. Contrariness *ψ* was chosen to be two orders of magnitude less than *σ*, to represent its rarity, although we investigated values around this range. The unit ‘plants’ refers to the number of plants in a field.

parameter	description	value^a^	source
crop agronomy			
*h*	harvesting rate	0.003 day^−1^	Jeger *et al.* [32]
*µ*	replanting rate	0 plants day^−1^	see also van den Bosch *et al.* [33]
*g*	roguing rate	0 day^−1^	—
*N*	number of fields	1000	—
disease dynamics			
*β*_p_	infection rate of plant	0.007 vector^−1^ day^−1^	Mware *et al.* [34]
*γ*_p_	disease progression rate in plant	0.035 day^−1^	Mware *et al.* [34] and Mohammed [35]
*η*	reversion ratio	0	—
*ξ*	cutting selection	0%	—
*β*_v_	virus acquisition rate for vector	0.007 plants day^−1^	Mware *et al.* [34]
*λ*	rate of loss of disease by vector	1 day^−1^	Legg *et al.* [14] and Patil *et al.* [36]
*R*	reservoir host density	0	—
vector dynamics			
1/*α*	mean vector dispersal distance	150 m	Byrne [37] and Isaacs & Byrne [24]
*W*	vector population	200 individuals	Jeger *et al.* [32]
*ω*	vector natural death rate	0.12 day^−1^	Jeger *et al.* [32]
*m*	vector migration rate	0.04 day^−1^	Riis & Nachman [23]
*A*	attractive area of field	20 000 m^2^	—
trading behaviour			
*b*_c_	chance of a grower trading for planting material	50%	Njenga *et al.* [10] & Otim-Nape *et al.* [17]
*b*_s_	maximum trading partners	3	Rohrbach & Kiala [31]
*b*_l_	growers loyal to trading partners	0%	—
grower behaviour			
—	farmer group size	15–30 growers	A. Pariyo, 28 April 2014, personal communication
*r*_l_, *r*_t_, *r*_d_	probability of receiving information from farmer group, trade partners, extension worker (district scale)	40%, 40%, 20%	see Apok [8]
*θ*	responsiveness of growers	1.6	see table caption
*κ*	conformity/stubbornness of growers	0	see table caption
*ψ*	contrariness of growers	0.001	see table caption
cost			
*p*	relative selling price of yield of uninfected field	1	T. Omara 2014, personal communication
*c*_clean_, *c*_reuse_	relative cost of using clean planting material, planting material recycled through reuse or trade	0.25, 0	T. Omara 2014, personal communication
*ι*	usable percentage of infectious plant	30%	e.g. Gondwe *et al.* [38] and Hillocks *et al.* [39]

^a^Certain parameters included for generality in the model are set to zero to simplify testing of hypotheses.

## 3. Results

### 3.1. How to increase use of a clean seed system

**Figure 1.**
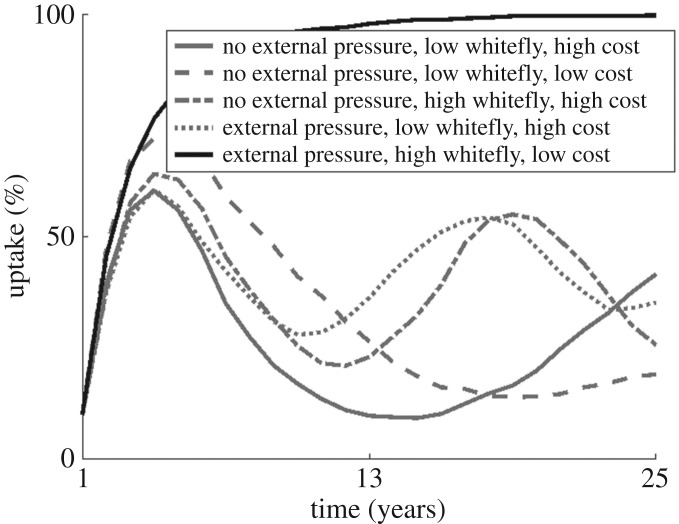
Low adoption of clean planting material over time in a system where there is no external disease pressure, reasonably high whitefly numbers and clean planting material is expensive (200 whitefly per plant, planting material cost = 1/4 maximum potential yield, solid grey line). External disease pressure (simulated through the immigration of infected whitefly from external sources at rate ***m*** or the introduction of a reservoir infected host at density 0.1, dotted line), higher whitefly numbers (500 whitefly per plant, dot-dashed line) or a low cost to clean planting material (planting material cost = 1/10 maximum potential yield, dashed line) alone are insufficient to promote high adoption. However, when combined, adoption approaches 100% (black line).

**Figure 2.**
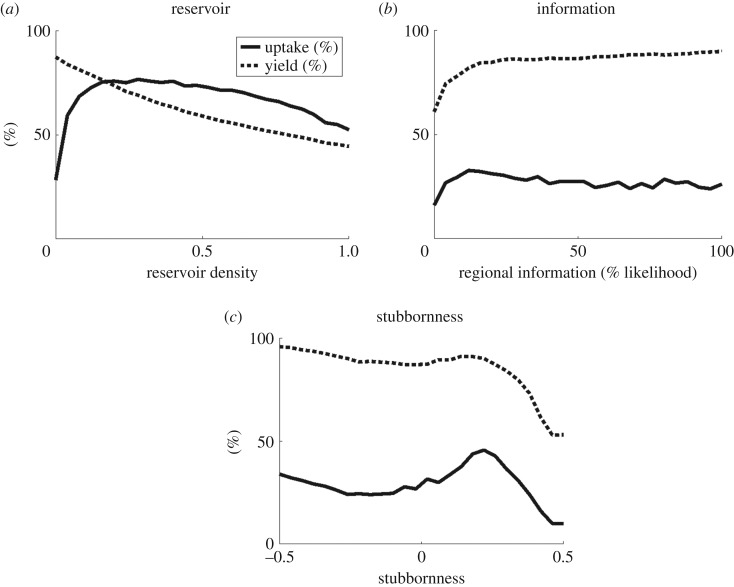
Change in average adoption (solid line) and yield (dashed line) with (*a*) density of a reservoir host, (*b*) likelihood of information being obtained at a regional scale, and (*c*) stubbornness of growers to change over 25 seasons.

Complete adoption of clean planting material by the entire grower population is never reached with our default parameter set (figure 1), as approaching ubiquity of adoption reduces disease to such an extent as to ensure clean planting material is unnecessary. However, if external disease pressure and whitefly numbers are particularly high, and the cost of clean planting material low, adoption can increase to very high levels as clean planting material becomes both necessary and readily available. In the absence of these factors, lower adoption, and oscillations in the adoption over time, are likely to occur. We examine a range of results, focusing on non-monotonic relationships here (figure 2). A summary of the effect of individual parameter values on adoption, yield and oscillations in both is outlined in table 2.

**Table 2.** Change in dynamics for increasing parameter values. Parameter values are grouped into those describing the agronomic system, the disease pressure, the clean planting material, the actions of growers and their behaviour. Varying these parameters affects the adoption of clean planting material and the percentage yield obtained, averaged over a period of 25 seasons, as well as the amplitude and frequency of oscillations and their dampening in adoption and yield. Increasing parameter values may increase or decrease these results monotonically (↗ or ↘ respectively), have no effect at all (→) or have a non-monotonic effect (↗↘, ↘↗ or ↘↗↘). Note that in all cases oscillations in adoption and yield are similarly affected by changes in parameter values.

parameter	adoption	yield	dampening	amplitude	frequency
casual	→	→	→	→	→
commercial	↗	→	↘	↗	↗
likelihood of trading	→	↘	↘	↗	↗
number of suppliers	↗	→	↘	↗	↗
disloyalty to suppliers	↗	↘	↘	↗	↗
initial disease pressure	→	→	→	↗	↘
number of whitefly	↗	→	↗	↘	↗
reservoir host	↗↘	↘	↗	↘	↘
cost of material	↘	↘	↗	↘	↗
tolerance of material	↘	↗	↗	↘	↘
resistance of material	↘	↗	↗	↘	↘
cleanliness of material	↘	↗	↘	↗	↘
roguing	↘	↗	↗	↘	↘
cutting selection	↘	↗	↗	↘	↘
information sources	↗	↗	↘↗	↗	↗
responsiveness	↘	↗	↘↗	↘	↗
stubbornness	↘↗↘	↘↗↘	↘↗	↗↘	↘
contrariness	↗	↗	↗	↘	↘

**Figure 3.**
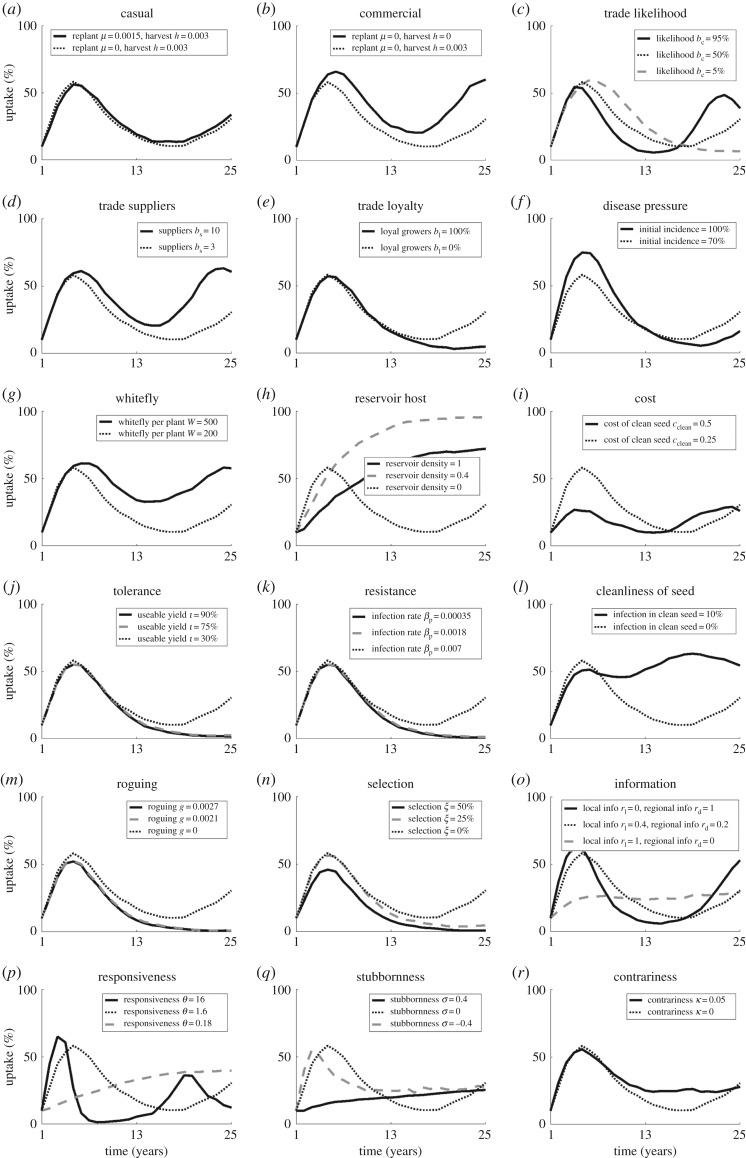
Effect of parameter changes on oscillations in adoption compared to the default parameter set (dotted line). Results show adoption for (*a*) a casual or (*b*) a commercial system, and for an increasing (solid line) or decreasing (dashed line) parameter values for (*c*) the likelihood of trade occurring, (*d*) the number of suppliers, (*e*) the number of growers disloyal to suppliers, (*f*) the initial incidence of disease, (*g*) the number of whitefly per plant, (*h*) the density of a reservoir host, (*i*) the cost of planting material, (*j*) the tolerance to disease of the planting material, (*k*) the resistance to disease of the planting material, (*l*) the incidence of disease in the planting material, (*m*) the rate of roguing, (*n*) the likelihood of successfully selecting out infectious cuttings, (*o*) the likelihood of growers obtaining regional-scale information, (*p*) the responsiveness of growers, (*q*) the stubbornness of growers or (*r*) the contrariness of growers.

**Figure 4.**
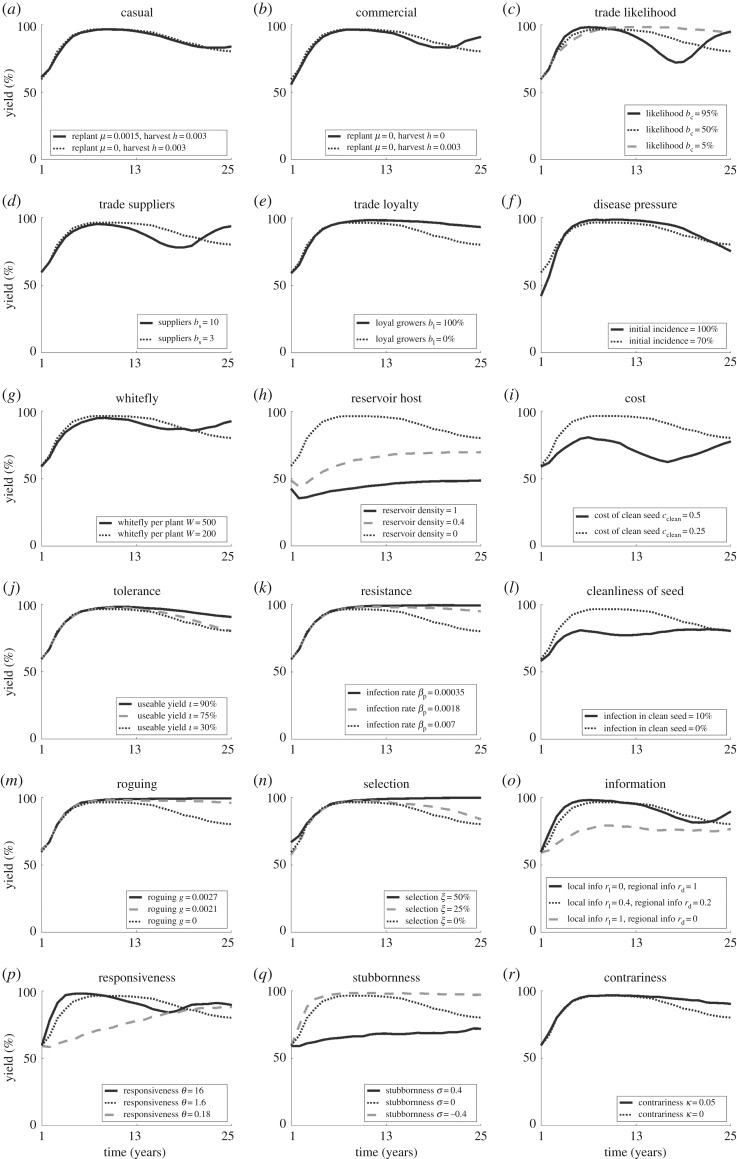
(*a*–*r*) Effect of parameter changes on oscillations in yield compared to the default parameter set (dotted line) as in figure 3.

*Agronomic system:* the degree of coordination and synchronization in planting and harvesting affects adoption and disease levels. Commercial systems (involving seasonal planting and harvesting) are likely to see higher levels of adoption than casual or subsistence systems (figure 3*a*,*b*), as seasonal harvesting ensures that more plants are in the field for longer, increasing their chances of infection. If there are high levels of trade in a region (a reasonable likelihood of trade with many, varying suppliers) we are likely to see an increase in adoption and a decrease in yields as the pathogen is able to spread more rapidly resulting in increased disease (figures 3*c*–*e* and 4*c*–*e*).

*Disease pressure*: areas with increased overall disease pressure are likely to incur lower yields and higher adoption. However, high initial disease pressure only affects the yield and adoption in the short term (figures 3*f* and 4*f*), after which disease pressure and therefore adoption equilibrate to similar levels regardless of the initial disease pressure. Additionally, the adoption varies to compensate for whitefly numbers, resulting in little change to yield (figures 3*g* and 4*g*). The presence of an alternative, reservoir host also only increases adoption (due to increased disease) up to a given density, above which adoption declines as the reservoir provides too large a source of infection for effective control to be worthwhile (figures 2*a* and 3*h*).

*Clean planting material*: costly clean planting material is less likely to be used as it begins to outweigh the benefit of its use, so that disease increases and yields decline (figures 3*i* and 4*i*). On the other hand, improved planting material, which is either tolerant or resistant and has low levels of infection, may increase yield but with a subsequent decrease in adoption (figures 3*j*–*l* and 4*j*–*l*) as growers' assessment of disease risk declines. However, the planting material must be highly improved, or it may lead to very little change in yield as the adoption varies to compensate. In such cases, an extreme reduction in disease means that growers no longer see the need for clean planting material, allowing for disease persistence and subsequent re-emergence.

*Grower activity*: practices that remove infected plants (roguing and selection) reduce adoption as they provide an alternative method of disease reduction, but have no effect on yields unless the rate is high enough to near-eradicate the disease, when yields rapidly increase (figures 3*m*–*o* and 4*m*–*o*). If growers are able to access information from a number of sources (i.e. have an increased likelihood of obtaining regional-level information) both adoption and yield increase (figures 3*o* and 4*o*) as growers across the region are able to assess the threat of disease. However, the increase in both adoption and yield is primarily noticeable for very low levels of regional information, above which there is less effect (figure 2*b*); there needs to be some access to global information, so that assessment of disease risk and clean planting material use is not confined to affected areas only, but this need not be ubiquitous as local dynamics are still important in determining risk of infection.

*Grower behaviour*: a high degree of responsiveness means that growers react quickly to the presence of disease, slowing disease dispersal and therefore reducing the need for adoption of clean planting material and increasing the yield (figures 3*p* and 4*p*). In terms of adoption, there is an optimum level of stubbornness of growers; above and below this optimum the adoption decreases, although with an additional increase in adoption again as growers begin to conform (figure 2*c*). This represents the balance in rapidity of response; if growers are too stubborn the majority will persist with their current strategy of non-engagement with clean planting material, while if they conform too readily the majority will quickly take up clean planting material, but then just as rapidly abandon it until an equilibrium number of users is reached. Yield, however, is highest for conforming growers, with a lower peak at a medium level of stubbornness as growers are quicker to follow the example of their neighbours and use clean planting material. Contrariness of growers increases both adoption and yield, as growers continue to use the system when it is unnecessary (figures 3*r* and 4*r*).

### 3.2. How to reduce oscillations

We have chosen to present only a 25-year span here, as this is both more relatable for practitioners and more realistic in terms of a period of interest. Increasing the simulation time does not change our qualitative conclusions about what promotes oscillations (although quantitative differences exit).

*Agronomic system*: an abundance of commercial growers is likely to see increased oscillations compared with a population comprised of casual or subsistence growers (figures 3*a*,*b* and 4*a*,*b*), as the effect of clean planting material through replanting becomes seasonal rather than continuous. Higher levels of trade result in more frequent, higher amplitude oscillations occurring as both the pathogen and clean planting material are disseminated quickly through the region (figures 3*c*–*e* and 4*c*–*e*).

*Disease pressure*: the initial pressure has little effect on oscillations except to increase the amplitude for very high initial incidence (figures 3*f* and 4*f*), as the dynamics quickly account for the initial state. However, consistent high disease pressure through high numbers of the vector or an alternative reservoir host leads to low amplitude, dampening oscillations as there is always disease present, so always a need for clean planting material (figures 3*g*,*h* and 4*g*,*h*).

*Clean planting material*: increasing the cost of clean planting material leads to reduced amplitude and dampening of oscillations as very few growers use the system (figures 3*i* and 4*i*). In comparison, tolerant or resistant planting material may dampen oscillations, decreasing the frequency and amplitude as plants are less affected by the disease (figures 3*j*,*k* and 4*j*,*k*). In contrast, infection in planting material leads to frequent, low amplitude oscillations with significant dampening, due (as with the presence of a reservoir host discussed above) to the constant presence of disease (figures 3*l* and 4*l*).

*Grower activity*: practices removing infected plants also dampen oscillations, lowering the amplitude and frequency (figures 3*m*,*n* and 4*m*,*n*) as disease is removed from the region through other means than clean planting material. However, if the disease is not completely eradicated it may return rapidly as control is no longer practiced. At the same time, very high or very low average numbers of information sources (due to high or low likelihood of obtaining regional level information) lead to dampening of oscillations (figures 3*o* and 4*o*), where growers make decisions based on either regional dynamics (dampening oscillations due to the interactions of different groups of growers) or very local dynamics (dampening oscillations as local trends have no opportunity to spread).

*Grower behaviour*: high or low responsiveness of growers leads to dampening of oscillations in adoption and yield; in the case of high responsiveness, growers react quickly to change before large population-wide trends develop, while for low responsiveness growers respond much slower to change than the disease dynamics, allowing for equilibration in the infection levels (figures 3*p* and 4*p*). Similarly, a high frequency of highly stubborn or conforming growers dampens oscillations (figures 3*q* and 4*q*). Contrariness also dampens oscillations in adoption and yield (figures 3*r* and 4*r*) as growers fail to respond to oscillations in disease dynamics.

### 3.3. How to implement an intervention

**Figure 5.**
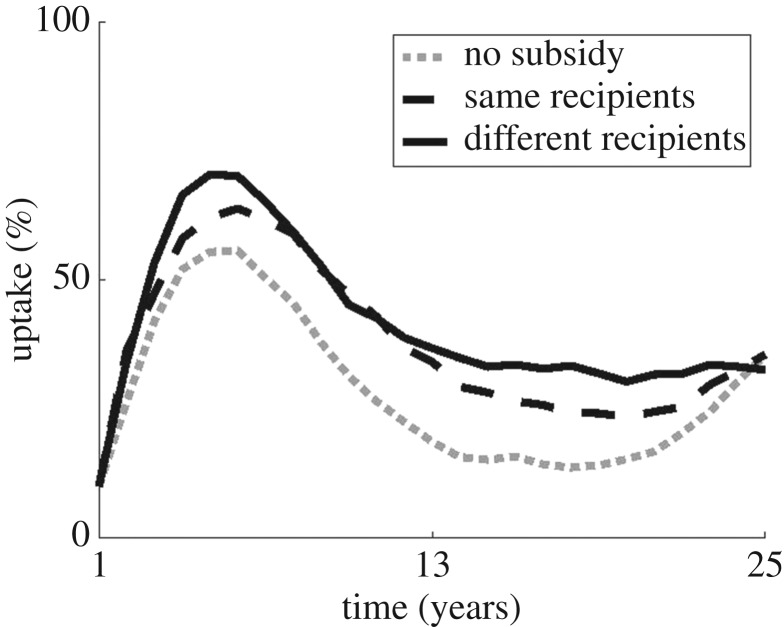
Different approaches to distributing subsidized clean planting material to 10% of growers for free, and the effect on adoption in the population as a whole. Distribution occurs at random throughout the district, either to different growers each season (black solid line) or to the same growers each season (black dashed line), compared to a case with no subsidy (grey dotted line). Results are similar if distribution focuses on communities of growers, as opposed to dispersed individuals.

A subsidy of free clean planting material to a given group of growers increases adoption (figure 5) as more growers are exposed to the benefits of the material, and has the most effect if distributed to varying recipients as suggested previously (see [16]). This is likely because the majority of growers in the region are repeat users, using the system for more than one season, so any overlap in past and present recipients of a subsidy is wasteful. Indeed, varying the recipients of the subsidy has the additional incentive of dampening oscillations in both yield and adoption with a small but consistent effect. However, any clustering of these recipients makes very little difference to adoption (not shown), contrary to previous findings (see [16]).

## 4. Discussion

Most epidemic models do not account for grower behaviour explicitly: for some exploratory exceptions see [4–6,40]. In this paper, we have built on a well-established framework for a parsimonious epidemic model with dual sources of infection (via vectors and replanting) and introduced simple elaborations that allowed us to gain insight into some of the consequences of grower choices on epidemic dynamics. Future work will refine the socio-economic aspects of the model as more empirical data become available to inform the choice of framework.

We note firstly that grower behaviour is, as expected, highly likely to impact disease spread through the adoption of clean planting material [5] and we are unlikely ever to see complete disease prevention (see [41]). Indeed, if external disease pressure or a reservoir host (although in reality this appears unlikely to be important) is present at sufficiently high levels, the release of disease resistant clean planting material may be necessary to control the disease at all. However, growers in areas with high disease pressure are more likely to show interest in clean planting material, as we might expect (see [8]), which allows for disease persistence in low pressure areas. Additionally, oscillations in adoption and yield are highly likely, as witnessed in other agronomic systems ([4], and hearsay from varieties resistant to cassava mosaic disease in Uganda at the turn of the century). In particular, areas with many commercial plantings (rather than casual or subsistence growers) are highly likely to succumb to large oscillations in adoption, even when differences in behaviour are not considered. One aspect not covered here is the relative importance of the crop to grower, compared with their reliance on other crops, which will also affect whether they are likely to consider disruptive management practices.

In terms of the planting material, competitively low pricing can significantly increase use; indeed, there is a threshold (where the cost of planting material is equal to the minimum yield that can be obtained from a fully infected field) above which growers are unlikely to use the planting material at all. We assume that the cost of the planting material also implicitly includes a cost in terms of a reduction in quality or desirability of the variety for growers, who may prefer local landraces. We note on the other hand that seed companies in other agronomic systems often bundle a number of desirable traits together (similar here to increasing the reward that using the clean seed system is perceived to give), as well as varying the cost of planting material, in order to avoid oscillations in adoption, an aspect that is not explicitly considered in the current analyses [4]. Planting material that is partially resistant will increase yields, but in reality may require replenishment as these yields decline with repeated recycling, an aspect we do not consider here. On the other hand, planting material that is tolerant will maintain high yields, but will also result in a build-up in inoculum pressure that may affect other varieties. However, infection in planting material will, economically at least, not dissuade growers from its use, although work on opinion dynamics by A. Milne (personal communication, 12 May 2016) suggests that trust in a control strategy is vital for its success.

At the same time, over-investment in the spread of information on success of the clean seed system appears to be unnecessary (see also [7], but see [21]); word of mouth between growers may often be sufficient, with little additional reward in terms of yield for any increase in information distribution. Reducing the dissemination of information may also slow grower reactions to change, decreasing oscillations (see also [4]). This implies that an optimal amount of information disseminated will lead to high adoption and yield, but with a low chance of oscillations occurring.

Education, influencing grower behaviour, is also important (e.g. [21]). Education affects responsiveness and conformity, as well as potentially improving agronomic practices and affecting the management of additional pests and diseases not considered here. Responsiveness among growers reduces adoption while increasing yield, and also dampens oscillations (see also [4]). However, so does very low responsiveness. Ideally, growers should assess their particular situation individually (i.e. not be too conformist, although if conformism is particularly high that can also increase adoption) but also be reasonable (not be too stubborn), where it may be key to stress the folly of hasty decisions and the wisdom of a certain degree of persistence (ensuring some level of stubbornness, see also [4]). However, the behaviour described above may also lead to oscillations. This may reflect the ‘prisoner's dilemma’ faced by growers undertaking potentially costly action to benefit the community. Surprisingly, contrariness of growers leads to high adoption with dampened oscillations, as growers persist with an unnecessary strategy.

## 5. Conclusion

Our model identifies five key aspects to consider when implementing a clean seed control strategy. Importantly, it has been necessary to consider grower behaviour here; in the absence of grower choice the model tends towards stable steady states (see also [16]). However, when we allow for the reaction of growers to this, cycles in adoption and incidence can result. It is vital to bear these cycles in mind when planning disease control.

Firstly, high disease pressure and aspects that lead to this are likely to encourage individuals to engage with a control strategy. However, if disease pressure is too high or persistent, individuals may be discouraged from engagement as control has little effect. Aspects that lead to rapid dispersal of the pathogen may also lead to oscillations in engagement, although consistent disease pressure dampens these oscillations as there is a constant demand for control.

Secondly, improvements in clean planting material and field management can be effective in reducing disease, although also decreasing engagement as control is no longer necessary (which may in turn be useful if only a limited supply of clean planting material is available). However, these improvements must be of a sufficient standard or they risk leading to near but not complete control of disease, at which point individuals cease to engage with the control strategy allowing for re-emergence of the disease.

Thirdly, high levels of information need not be disseminated in order to have an effect on engagement, although there must be some chance of individuals gaining a broad view of disease risk. If this chance is high, but not guaranteed, it may lead to oscillations in engagement.

Fourthly, education of individuals is effective in promoting rapid response to changes in disease pressure, although if this response is not sufficiently rapid it may also promote oscillations in engagement and disease. However, we should not be discouraged by a certain degree of stubbornness or contrariness in the population, which may increase engagement over time as well as dampening oscillations in this.

Finally, the introduction of some level of ‘free’ control can have a marked positive effect on both engagement and disease reduction. This control need not be associated with a complex, coordinated approach to achieve high impact. However, in order to have maximum effect, as well as to dampen oscillations in engagement, it is important that the control reach different individuals over time, rather than the same select group consistently.

## Data accessibility

The Matlab code for simulations described above can be found at http://doi.org/10.5281/zenodo.556986.
